# Tick Salivary Compounds for Targeted Immunomodulatory Therapy

**DOI:** 10.3389/fimmu.2020.583845

**Published:** 2020-09-23

**Authors:** Hajer Aounallah, Chaima Bensaoud, Youmna M’ghirbi, Fernanda Faria, Jindr̆ich Chmelar̆, Michail Kotsyfakis

**Affiliations:** ^1^Institut Pasteur de Tunis, LR19IPTX, Service d’Entomologie Médicale, Université de Tunis El Manar, Tunis, Tunisia; ^2^Innovation and Development Laboratory, Innovation and Development Center, Instituto Butantan, São Paulo, Brazil; ^3^Institute of Parasitology, Biology Centre, Czech Academy of Sciences, České Budějovice, Czechia; ^4^Department of Medical Biology, Faculty of Science, University of South Bohemia in České Budějovice, České Budějovice, Czechia

**Keywords:** tick saliva, salivary glands, host immunity, immunomodulation, drug discovery

## Abstract

Immunodeficiency disorders and autoimmune diseases are common, but a lack of effective targeted drugs and the side-effects of existing drugs have stimulated interest in finding therapeutic alternatives. Naturally derived substances are a recognized source of novel drugs, and tick saliva is increasingly recognized as a rich source of bioactive molecules with specific functions. Ticks use their saliva to overcome the innate and adaptive host immune systems. Their saliva is a rich cocktail of molecules including proteins, peptides, lipid derivatives, and recently discovered non-coding RNAs that inhibit or modulate vertebrate immune reactions. A number of tick saliva and/or salivary gland molecules have been characterized and shown to be promising candidates for drug development for vertebrate immune diseases. However, further validation of these molecules at the molecular, cellular, and organism levels is now required to progress lead candidates to clinical testing. In this paper, we review the data on the immuno-pharmacological aspects of tick salivary compounds characterized *in vitro* and/or *in vivo* and present recent findings on non-coding RNAs that might be exploitable as immunomodulatory therapies.

## Introduction

The vertebrate immune system is a sophisticated and highly developed network of cells, tissues, and organs that together identify and neutralize foreign and endogenous threats. Immunodeficiencies represent a breakdown in these highly organized processes caused by a lack or dysfunction of specific immune cell subpopulations or soluble effectors (1). Immune disorders also arise due to excessive immune responses, such as in allergic reactions or autoimmunity (2), usually as a result of dysregulation or overexpression of specific cytokines and their related immune signaling pathways (3). The human immune system is also affected, and often weakened, by stress, malnutrition, and age-related changes, which increase susceptibility to infectious diseases and other pathologies such as cancer (4–6). Current therapies for immune-related illnesses usually have side-effects or suboptimal efficacy (7, 8), leading to efforts to identify alternative therapies that might naturally inhibit and/or modulate specific immune system targets without significant off-target effects (9), including bioactive molecules derived from natural sources (10). Compounds with therapeutic potential have traditionally been extracted from plants (phytotherapy) (11), but other sources have included scorpion and snake venom (12, 13) and arthropods (14). However, substances extracted from natural sources and applied in practice remain limited, and their clinical utility is hampered by the risk of contamination with impurities (15). More positively, drug discovery has been assisted by the development of new protein production methods using various expression systems (16–18), and advances in new technologies such as next-generation sequencing (NGS) and mass spectrometry (MS) have revolutionized screening for novel natural substances (19, 20).

Tick salivary glands are now recognized as a rich source of pharmaco-active molecules (21). Tick saliva contains a rich cocktail of bioactive molecules including protein and lipid derivatives with a remarkable binding affinity, avidity, and selectivity for their targets in various host defense systems (22). Ticks are obligatory hematophagous that, in order to feed, must overcome the evolutionarily sophisticated immune defense systems of their vertebrate hosts. To achieve this, they secrete a wide variety of molecules in their saliva with immunomodulatory, anti-inflammatory, anti-clotting, and anti-platelet effects (23, 24).

Despite the identification of a large number of bioactive tick salivary molecules, their investigation for therapeutic purposes remains in its outset. Only a limited number of tick salivary compounds, mostly proteins, have been tested pre-clinically (25, 26). Here we discuss the literature on therapeutically valuable tick salivary molecules with function(s) known to be directly related to host immune responses. In doing so, we identify the most promising salivary candidates with drug development potential, including newly discovered non-coding (nc) RNAs.

## Tick Saliva Targets at the Tick-Host Interface

**FIGURE 1:**
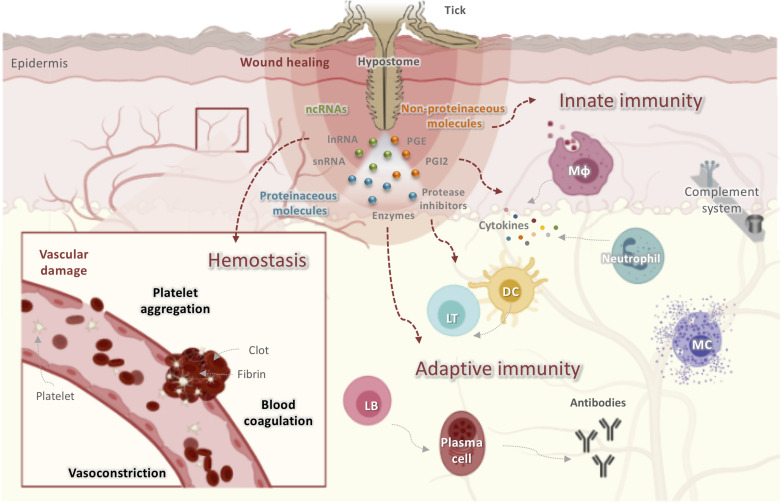
Obstacles faced by ticks at the host-tick interface when taking a blood meal. Ticks initiate feeding by inserting their hypostomes into host skin, resulting in tissue and vascular damage. The host has developed several mechanisms to prevent blood loss including activating hemostasis, innate and adaptive immunity, the complement pathway, and inflammatory responses leading to wound healing and tissue remodeling, all of which disrupt tick feeding. Ticks, in turn, secrete saliva at the bite site that contains proteinaceous molecules (enzymes, lipocalins, protease inhibitors, etc.), non-proteinaceous molecules (prostaglandins, prostacyclins), and ncRNAs (miRNAs and lncRNAs). These molecules display anticoagulatory, antiplatelet, vasodilatory, anti-inflammatory, and immunomodulatory activities to counter host reactions and to guarantee a successful blood meal. DC, dendritic cells; LT, lymphocyte T; LB, lymphocyte T; Mφ, macrophage.

Ticks are obligate hematophagous ectoparasites of amphibians, reptiles, birds, and mammals that cause host blood loss and skin damage (27). Compared to other blood feeders, tick feeding behavior is unique. Hard ticks discretely and solidly attach to their hosts for several days to weeks to complete their blood uptake (28), with the skin representing the main interface where tick salivary compounds meet host defense systems (29, 30) (Figure 1). Furthermore, the skin is the site of multiplication and persistence of several tick-borne pathogens (31, 32). In the local environment of the skin, the tick-host molecular interaction can be viewed as a competition between host defense mechanisms against the ectoparasite and tick evasion strategies (30). To ensure an uninterrupted blood meal, ticks have developed myriad strategies to overcome the complex homeostatic and immune responses that are raised against them (22). As long-term pool feeders, they create an immunologically privileged micro-environment in the host’s skin, in which they secrete an impressive mixture of proteins, peptides, and non-peptide molecules (Figure 1), thereby modulating wound healing, hemostasis, inflammation, and both the innate and adaptive immune responses (33). These molecules interfere with various host molecules including enzymes, cytokines, complement components, antibodies, cell signaling molecules, and immune cell receptors (33).

Hemostasis is triggered within seconds of tissue damage and is the product of the triad of blood coagulation, platelet aggregation, and vasoconstriction, a process mainly controlled by serine proteases (34). The anti-hemostatic properties of tick salivary secretions are reviewed elsewhere (23, 35). In addition to hemostasis, complement components and inflammatory mediators are also initial tick saliva targets (36, 37). When tick mouthparts are inserted into the skin, pre-positioned sentinel leukocytes of the epidermis and dermis, including mast cells (MCs), eosinophils, dendritic cells (DCs), and macrophages, as well as keratinocytes and endothelial cells, are activated by mediators released from damaged skin cells or expressed by pathogens transmitted by ticks (38). The resident macrophages release pro-inflammatory chemokines and cytokines including interleukin-8 (IL-8), tumor necrosis factor (TNF), and IL-1β (38). These chemoattractants recruit blood-borne innate immune cells such as neutrophils and monocytes to the bite site, which intensify the stimulation of local and infiltrating innate immune cells (30, 39). Monocytes secrete growth hormones that induce fibroblast proliferation and extracellular matrix deposition, thus contributing to wound healing (39). Given their parasitic lifestyle, ticks must overcome innate immunity during their primary infestation and both innate and adaptive immunity during secondary or subsequent infestations (22). Following tick feeding, tick-derived antigens may be presented to naïve B and T cells by activated DCs that have acquired foreign antigens and have migrated to skin-draining lymph nodes (40). Both the humoral and cellular branches of host adaptive immunity are activated, thereby resulting in the generation of antigen-specific antibodies and T lymphocytes (40). In subsequent infestations, activated memory T and B lymphocytes secrete cytokines and produce specific antibodies that target tick salivary or mouthpart-derived antigens in an effort to reject the tick (41).

## Impact of Tick Salivary Compounds on Host Immune Responses

The complex mixture of tick salivary compounds specifically and selectively targets host immune reactions, subverting the rejection and death of the tick (24). This specificity and selectivity may therefore be exploited for diseases in which dysfunction of the same host immune reactions is implicated in their pathogenesis, for instance the innate immune system in Sjögren’s syndrome (42), adaptive immunity in Tregopathies (43) and autoimmune encephalomyelitis (44), and complement activation in local and/or systemic inflammation, tissue damage, and disease (45).

Some tick salivary components have immune system specificity at different steps of immune recognition (21). Despite this specificity, tick salivary component targets often show redundancy at the molecular, cellular, and functional level (i.e., may be targeted by more than one tick salivary molecule) (29). Furthermore, several tick salivary compounds are pleiotropic, targeting both hemostatic and immune system components (29). For instance, Salp15, a multifunctional protein, was shown to bind specifically to dendritic cell, inhibit CD4 + T cell activation and proliferation, and block the liberation of interleukin 2 (IL-2) (46). Apart from its immunomodulatory activities, it has also been described to modulate host coagulation (47). This pleiotropicity could be problematic in targeted immunotherapy, which relies mainly on single target drugs and is often considered as a serious limitation in drug conception. Nevertheless, this issue does not exclude the possibility of using these salivary components in other therapeutic applications. Multi-target drugs, also known as multifunctional drugs or network therapeutics are more suitable as potential therapeutic solutions in diseases of complex etiology, such as Alzheimer’s disease, Parkinson’s disease, and neglected tropical diseases (48). Single-target drugs, although highly selective and specific, may not necessarily have better efficacy in these cases (49). Indeed, multi- target drugs tend to be beneficial to face complex disorders, multifactorial health conditions and drug resistance issues (50, 51). Some studies trend to changing paradigm from “one target one ligand” toward “multi-target” as they does not provide a complete solution for multifactorial diseases (49). Therefore, tick salivary compounds with broad-spectrum targets might be useful in these diseases mediated by multiple processes.

The immunomodulatory properties of whole tick saliva and/or salivary gland extracts were previously reviewed by Kotál and colleagues (52), and some comprehensive and recent reviews have focused on the composition and role of saliva in tick feeding and tick-host-pathogen interactions (21, 53, 54). These articles have tended to focus on proteinaceous salivary component families including lipocalins, protease inhibitors such as Kunitz-type domains containing proteins, serpins, and cystatins, metalloproteases, basic tail secreted proteins, small peptide inhibitors, and some protein families unique to ticks. The potential of non-peptide molecules, lipid derivatives, and the recently discovered ncRNAs are rarely mentioned. This review therefore focuses on immunomodulatory substances that have been shown to target both the innate and adaptive host immune systems in *in vitro* and/or *in vivo* animal models of human diseases and in doing so we identify salivary candidates with promise for targeted immunotherapy. A limited number of salivary molecules are progressing in preclinical trials. This includes Amblyomin-X, Ir-CPI, TAP, OmCI, and Ra-HBP, among others. Most of these molecules have been described as anti-hemostatic and anti-tumor candidates (55). Only OmCI and Ra-HBP target immune responses, therefore, their potential applications will be discussed later in this review. Amblyomin-X, a Kunitz-type protease inhibitor, is profoundly exploited in pre-clinical testing and is under development for cancer treatment (26). It selectively induces apoptosis in tumor cells and promotes tumor reduction *in vivo* in melanoma animal models and reduce metastasis and tumor growth in *in vivo* experiments (56). In its pre-clinical evaluation, this protein was proven to significantly decrease lung metastasis in a mice orthotopic kidney tumor model (25). More interestingly, Amblyomin-X does not seem to cause any mortality; and symptoms of toxicity were subtle, reversible, and seen only at higher doses, thus demonstrating a safety profile for injection in mice. More recently, it was investigated on Amblyomin-X-treated horse melanomas showing significant reduction in the tumor size (57). Ir-CPI (*Ixodes ricinus* contact phase inhibitor), an antithrombotic protein, has proven its effectiveness in inhibiting the contact phase of the coagulation cascade in preclinical trials, preventing clotting in catheter and arteriovenous shunt rabbit models during Cardiopulmonary Bypass (58). TAP, a Kunitz domain protease inhibitor from *Ornithodoros moubata*, has been described as an anticoagulant candidate, showing promising results in *in vivo* models of venous and arterial thrombosis (59). Its efficacy on blood coagulation has been approved in preliminary preclinical experiments; however, it has never been inquired in humans mainly due to its antigenicity (59).

### Innate Immune Responses

**FIGURE 2:**
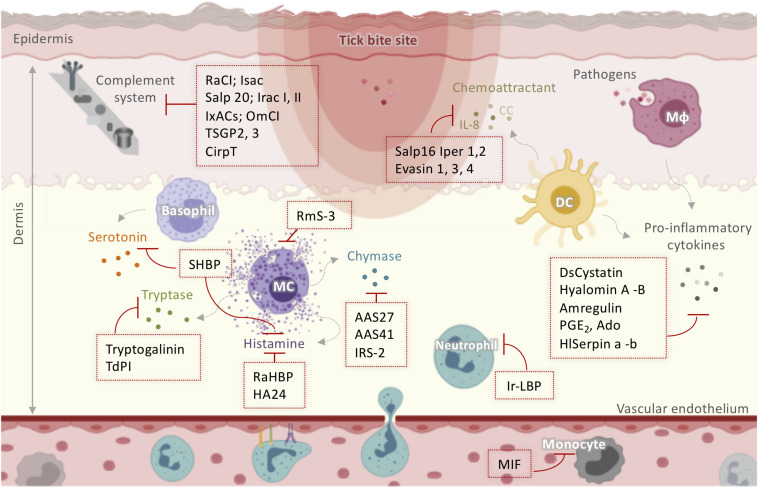
Schematic representation of tick salivary molecules implicated in the modulation of the complement system and innate immunity. Activated resident cells in the dermis stimulate host awareness of injury and removal of the feeding ticks. Tick saliva neutralizes itch and pain through salivary components that sequester histamine and/or serotonin (such as SHBP, Ra-HBPs, and HA24) or modulate MC function (such as RmS-3). TdPI and Tryptogalinin inhibit tryptase released by MCs. AAS27, AAS41, and IRS-2 inhibit chymase liberated by MCs. Neutrophil and monocyte recruitment is suppressed by MIF and Ir-LBP. Moreover, ticks manipulate the host cytokine network by inhibiting cytokines and chemokines using Salp16 Iper 1,2; Evasin 1, 3, 4; DsCystatin; Hyalomin-A, –B; Amregulin; PGE_2_; Ado; and HlSerpin-a, –b. Several anti-complement molecules have been identified in tick salivary glands including RaCI; Isac; Salp 20; Irac I, II; IxACs; OmCI; TSGP2, 3; and CirpT. AAS: *Amblyomma americanum* serpin; DC: dendritic cells; IL: interleukin; Irac: *I. ricinus* anticomplement; IRS-2: *I. ricinus* Serpin-2; Isac: *I. scapularis* salivary anticomplement; IxACs: *Ixodes* anticomplement proteins; MIF: macrophage migration inhibitory factor; Mφ: macrophage; OmCI: *O. moubata* complement inhibitor; PGE_2_: prostaglandin E2; Ra-HBPs: *R. appendiculatus* histamine-binding proteins; RmS: *Rhipicephalus microplus* serpin; Salp: salivary protein; SHBP: serotonin- and histamine-binding protein; TdPI: tick-derived protease inhibitor.

The host innate immune response forms the first and immediate line of defense to tick attachment (52). Activated resident cells in the cutaneous barrier including MCs, macrophages, and DCs stimulate host awareness to the injury followed by removal of feeding ticks (Figure 2).

#### Itch and Pain

Mast cells and basophils degranulate to release soluble mediators such as histamine and serotonin, which cause local itch and pain at the infestation site (60). However, ticks alleviate itch and pain through salivary components that sequester histamine and/or serotonin or modulate MC function. RmS-3, a novel serpin extracted from the salivary glands of *Rhipicephalus microplus* (61), has been shown to modulate MC function by inhibiting chymase and vascular permeability in acute inflammation (Figure 2). The hard tick *Rhipicephalus appendiculatus* uses the histamine-binding proteins Ra-HBPs (in the lipocalin family) to bind histamine with high affinity during early feeding (62). Different Ra-HBPs were identified in male and female ticks: male-specific histamine-binding salivary protein Ra-HBP (M) and two female-specific histamine-binding salivary proteins RaHBP(F)-1,2 (Figure 2). Ra-HBP2 sequestered two histamine molecules with different affinities, emphasizing its therapeutic potential by targeting multiple effectors (63). Another lipocalin showing histamine binding capacity, HA24, was identified in the salivary glands of *Hyalomma asiaticum* (Figure 2) (64). *In vitro* and *in vivo* histamine binding assays showed that recombinant HA24 bound specifically to histamine in a dose-dependent manner and relieved allergic asthma in mice (64). SHBP (serotonin and histamine-binding protein), isolated from *Dermacentor reticulatus* (65), simultaneously interfered with the activity of both serotonin and histamine (Figure 2).

In addition to biogenic amine production, MCs contribute to the inflammatory process by releasing a wide range of highly bioactive effectors after degranulation, tryptases being the most abundant (66). Tryptases are implicated in the pathogenesis of allergic inflammatory diseases, cardiovascular disease, lung fibrosis, and even cancer, so their inhibition may be useful therapeutically (66, 67). Interestingly, tick-derived protease inhibitor (TdPI) was identified in the salivary glands of *R. appendiculatus* and was shown to suppress the activity of human β-tryptase and trypsin and, to a lesser extent, plasmin (68). TdPI accumulates in the cytosolic granules of mouse MCs, presumably blocking the autocatalytic activation of tryptase, thereby suppressing inflammation in the host animal’s skin (Figure 2) (69). Tryptogalinin, an *Ixodes scapularis* salivary Kunitz-type protein, was also found to inhibit β-tryptase (Figure 2) and other MC serine proteases such as α-chymotrypsin, plasmin, matriptase, and elastase and participated in inflammation and tissue remodeling (70). β-tryptases are MC-specific serine proteases with roles in inflammation that are used clinically as biomarker of MCs and their activation (67). Both tryptogalinin and TdPI could be engineered as highly specific pharmacological inhibitors of MC tryptases for the treatment of allergic inflammatory disorders like asthma (67–70).

#### Recruitment of Blood-Borne Innate Immune Cells

Within the first few hours after attachment, blood-borne leukocytes are recruited to the site of injury, triggered by a set of mediators including complement components, eicosanoids, chemokines, and cytokines (71). Neutrophil and monocyte recruitment has been shown to be strongly suppressed by tick salivary compounds. A homolog of the vertebrate macrophage migration inhibitory factor (MIF) was identified in *Amblyomma americanum* (72, 73) and *Haemaphysalis longicornis* (74). In both ticks, functional *in vitro* assays revealed that MIF inhibited the migration of human monocytes, suggesting that it might decrease monocyte recruitment *in vivo*. Ir-LBP from *I. ricinus* inhibited neutrophil chemotaxis and activation *in vitro* by binding specifically and with high affinity to leukotriene B4, an important inflammatory mediator (75). Ir-LBP was also shown to inhibit inflammatory responses in rabbits by decreasing the number of neutrophils located at the tick bite site (75). Therefore, Ir-LBP may have therapeutic use in inflammatory diseases or illnesses associated with increased leukotriene B4 production. Moreover, ticks employ salivary inhibitors of CXCL8 (IL-8) and CC chemokines to manipulate the host cytokine network. Salp16 Iper1 and Salp16 Iper2 (Figure 2), salivary proteins from *Ixodes persulcatus*, have been shown to have anti-IL-8 activity, thereby impairing neutrophil chemotaxis (76). A very recent review summarized the data on Evasins, which are secreted by hard ticks and bind to host chemokines to inhibit their activation of chemokine receptors (77). Of the Evasins, three from *Rhipicephalus sanguineus* (Evasin-1, -3, and -4) were selective for different chemokines (78). Evasin-1 bound to the CC chemokine members CCL3, CCL4, and CCL18 (78) and inhibited neutrophil, T cell, and macrophage migration and the production of inflammatory cytokines *in vitro* (78). Russo and colleagues demonstrated the high efficiency of Evasin-1 in reducing CCL3-induced influx of neutrophils in murine bleomycin-induced lung fibrosis (79). Evasin-1 has also been shown to reduce graft-versus-host disease in mice, which may be particularly useful in patients undergoing bone marrow transplantation (80). Moreover, the administration of recombinant Evasin-1 reduced skin inflammation and decreased mortality in mice deficient in the chemokine receptor D6, which renders then highly susceptible to inflammation (81). Evasin-3 is specific for the CXC chemokines CXCL8 and CXCL1 and was recently found to disrupt the interaction between CXCL8 and glycosaminoglycans and CXCR2 (Figure 2), which modulate neutrophil migration (82). Several studies have demonstrated the effectiveness of Evasin-3 in different neutrophil-dependent disease models. A single administration of Evasin-3 during mouse myocardial ischemia/reperfusion injury effectively reduced infarct size and decreased CXC chemokine-induced neutrophil recruitment (83). Evasin-3 also reduced atherosclerotic vulnerability to ischemic stroke in an *in vivo* murine model (84) and inhibited neutrophil-mediated pancreatic and lung inflammation in a mouse model of acute pancreatitis (85). Evasin-4 interacts with at least 18 CC chemokines (78). Similar to Evasin-3, Evasin-4 was effective against post-infarction myocardial injury and remodeling (86) and decreased the abundance of macrophages in the lungs without affecting the pancreas in the acute pancreatitis model (85). Due to its broad CC chemokine-binding spectrum, Evasin-4 is considered the most suitable candidate for therapeutic development (86). More recently, Evasin-inspired artificial peptides dramatically reduced inflammation *in vivo* by targeting multiple chemokines (87). These peptides might therefore provide a route to the development of new anti-inflammatory therapeutics for chronic autoimmune diseases such as rheumatoid arthritis and inflammatory diseases such as atherosclerosis. Moreover, the mechanism of action of these peptides suggests that they might also be useful in acute infectious diseases such as influenza or COVID-19, where exuberant cytokine responses are thought to be at least partially responsible for tissue injury (87).

#### Inflammation

Activated resident cells secrete several pro-inflammatory mediators, which initiate and then reinforce the local inflammatory process at the damaged site. Tick saliva controls inflammation by decreasing or increasing the secretion of pro- and anti-inflammatory cytokines, respectively (29). Two immunoregulatory peptides, Hyalomin-A and –B from *H. asiaticum asiaticum*, overcome host inflammation by modulating cytokine expression, inhibiting the secretion of pro-inflammatory TNF-α, MCP-1, and IFN-γ and stimulating the secretion of the immunosuppressant cytokine IL-10 (88). Amregulin from *Amblyomma variegatum* saliva was found to suppress the *in vitro* production of TNF-α, IL-1, IL-8, and IFN-γ in a dose-dependent manner (89). Hyalomin-A and –B and Amregulin significantly inhibited adjuvant-induced paw inflammation in mouse models *in vivo* (88, 89). HlSerpin-a and –b, novel serpins from the hard tick *H. Longicornis*, suppressed the expression of TNF-α, IL-6, and IL-1β from LPS-stimulated mouse bone marrow-derived macrophages (BMDMs) or mouse bone marrow-derived dendritic cells (BMDCs) (90). Three salivary gland serpins, AAS27 and AAS41 from *A. americanum* (91, 92) and IRS-2 from *I. ricinus* (93), inhibited inflammation by targeting chymase (Figure 2), an enzyme produced by activated MCs. IRS-2 also inhibited Cathepsin G, which is involved in tissue remodeling during inflammation and modulates the production of IL-6 by DCs, which subsequently impairs Th17 differentiation and maturation. DsCystatin from *Dermacentor silvarum* salivary glands was shown to impair the expression of the inflammatory cytokines IL-1β, IFN-γ, TNF-α, and IL-6 from mouse BMDMs (94), and the authors proposed that DsCystatin might be useful for the treatment of inflammatory diseases since it suppressed joint inflammation induced by complete Freund’s adjuvant (CFA) and *Borrelia burgdorferi* in a mouse arthritis model (95).

Another category of anti-inflammatory compounds are non-proteinaceous substances. It is important to point out that bioactive lipidic salivary component despite being abundant in the saliva of hard ticks; mainly prostaglandins PGE_2_ and PGF_2α_, only a few studies describe their pharmaceutical use (96, 97). Cannabinoids have also been detected in *Amblyomma* ticks and have been proposed to act as analgesic and anti-inflammatory compounds (98). However, these lipid derivatives have not been thoroughly investigated and require more rigorous investigation to assess their pharmacological interest. The two most documented non-proteinaceous compounds are purine nucleoside adenosine (Ado) and prostaglandin PGE_2_ (Figure 2) present in the saliva of *R. sanguineus* (99). Both compounds impaired the production of pro-inflammatory IL-12p40 and TNF-α and stimulated the release of anti-inflammatory IL-10 by murine DCs (99). In humans, Ado is a homeostatic regulator and a “danger signal” for cells and organs, since it is expressed during trauma or stress (100, 101). The therapeutic potential of Ado is well documented elsewhere (100–102). Ado is protective against several pathologies including inflammation and various forms of neuronal hyperexcitability and/or toxicity including hypoxia, seizures, and chronic pain (101). Similarly, PGE_2_ has pharmacological proprieties *in vitro* and *in vivo* associated with inflammation (103) and cancer (97) influencing cell proliferation, apoptosis, angiogenesis, inflammation, and immune surveillance (104). Thus, Ado and PGE_2_ derived from *R. sanguineus* saliva could be used for therapeutic purposes.

### Complement System

The complement system was originally regarded as a support to innate immunity against microbial invaders (45). More recently, complement has been recognized as having functions beyond microbial elimination such as clearance of immune complexes (105), complementing T and B cell immune functions (36), and tissue regeneration (106). Complement links the host innate and adaptive immune responses and is activated via three pathways (alternative, classical, and lectin) (31, 85). The excessive activation of complement components is responsible for a wide range of immune-mediated diseases (107) including autoimmune diseases, Alzheimer’s disease, schizophrenia, atypical hemolytic-uremic syndrome, angioedema, macular degeneration, and Crohn’s disease (108). Since the introduction of the first complement-specific drug, eculizumab, into the clinic, over 20 candidate drugs are now being evaluated in clinical trials and additional agents are in preclinical development (109). Several molecules with promising anti-complement activities were identified in tick salivary glands. For instance, Isac from *I. scapularis* (110) and its paralogs IRAC I and II from *I. ricinus* (111) specifically blocked binding of complement factor B to complement C3b *in vitro*, inhibiting the formation of the C3 convertase of the alternative pathway. Since convertases mediate nearly all complement effector functions, they are ideal targets for therapeutic inhibition (112). Selective inhibition of complement precursors and regulators is also of great therapeutic interest (109). Salp20 from *I. scapularis* (113) and IxACs from *I. ricinus* (114) inhibit the alternative pathway by binding properdin, a positive regulator of the pathway. Soft ticks have been reported to inhibit the classical complement pathway, with the lipocalins OmCI from *O. moubata* (115, 116) and TSGP2 and TSGP3 from *Ornithodoros savignyi* (117) specifically targeting C5 activation. The recombinant version of OmCI (also known as Coversin or rEV576) has been tested in several animal models of complement-mediated diseases and has shown efficacy in a clinical trial (115, 118, 119). By inhibiting C5 activation, OmCI significantly reduced excessive inflammatory reactions associated with severe forms of influenza virus A (IAV) in mice (116). In addition to its effect on C5, OmCI significantly decreased leukotriene B4 levels in septic pigs (120). In the same study, combined inhibition of complement C5 by OmCI and CD14 with anti-CD14 antibodies significantly attenuated inflammation, thrombogenicity, and hemodynamic abnormalities (115, 120), suggesting OmCI might be useful for the treatment of sepsis. OmCI was also effective in preventing experimental autoimmune myasthenia gravis induced by passive transfer in normal Lewis rats (118). RaCI, from *R. appendiculatus* salivary glands, bound human C5 and blocked C5a generation and membrane attack complex formation (121). RaCI exhibits cross-species reactivity, allowing it to be used in disease models to test therapeutic effectiveness. An OmCI and RaCI homolog, CirpT, is a new class of complement inhibitor identified in *Rhipicephalus pulchellus* saliva that binds C5 *in vitro* via a unique binding site that does not overlap with other known inhibitors (122). Eculizumab, an antibody against C5, is currently prescribed for diseases characterized by excessive activation of the alternative pathway, including paroxysmal nocturnal hemoglobinuria and atypical hemolytic uremic syndrome (123, 124). Despite its effectiveness, eculizumab is one of the most expensive drugs in the world (121). Nevertheless, these other proteins derived from tick saliva offer exciting prospects for C5-targeted therapeutics of complement-mediated diseases and could be alternatives to eculizumab. In addition to the alternative pathway, the lectin pathway is also targeted by the tick salivary lectin pathway inhibitor (TSLPI), identified from the salivary glands of *I. scapularis* (125). The lectin pathway is activated in an antibody and C1-independent manner, upon the interaction of Mannose-binding lectin (MBL) with hyper glycosylated pathogen-associated molecular patterns (PAMPs) on microbial surfaces (126). TSLPI was described to directly inhibit MBL complement pathway activation (125). Additionally, both TSLPI and its ortholog from *I. ricinus* were shown to protect *Borrelia burgdorferi* sensu lato from complement-mediated killing, by inhibiting MBL function (127, 128). MBL appears to influence the severity of several diseases including pancreatic ductal adenocarcinoma (PDAC) (129). During a Pancreatic Cancer, MBL is required for oncogenic progression, whereas inhibition of MBL by TSLPI could be protective against tumor growth.

### Acquired Immune Responses

**FIGURE 3:**
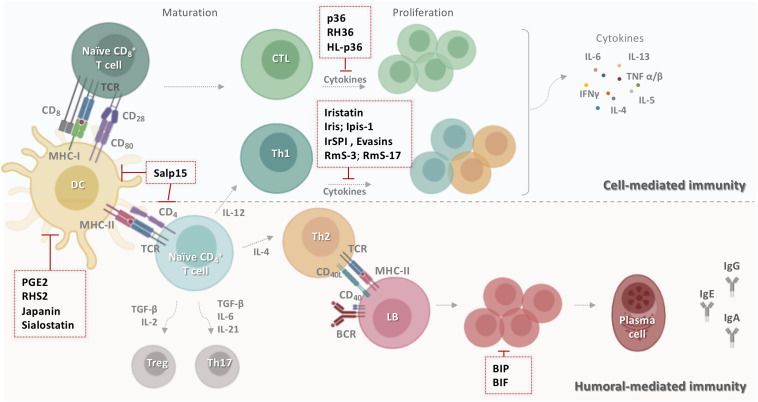
Schematic representation of tick salivary molecules targeting adaptive immunity. During the primary contact with ticks, dendritic cells present salivary antigens to lymphocytes, which trigger cell- and humoral-mediated responses. Some tick salivary molecules prevent the initiation of adaptive immunity by targeting DCs, including Salp15, PGE_2_, RHS2, Japanin, and sialostatin. Other compounds block the proliferation of lymphocytes and/or inhibit the production of T cell cytokines such as p36, RH36, HL-p36, Iristatin, Iris, Ipis-1, IrSPI, Evasins, RmS-3, and RmS-17. However, BIP and BIF disarm humoral host immunity by inhibiting B cell responses, including the production of specific anti-tick antibodies. BCR: B cell receptor; BIF: B cell inhibitory factor; BIP: B cell inhibitory protein; CD: cluster of differentiation; CTL: cytotoxic T lymphocyte; DC: dendritic cells; HL-p36: *Haemaphysalis longicornis* p-36; IFN-γ: interferon gamma; Ig: immunoglobulin; IL: interleukin; LB: lymphocyte B; MHC: major histocompatibility complex; PGE_2_: prostaglandin E2; RmS: *Rhipicephalus microplus* serpin; Salp: salivary protein; TCR: T cell receptor; TGF-β: transforming growth factor-beta; Th: T helper; TNF: tumor necrosis factor; Treg: regulatory T cell.

During the first encounter with ticks, antigen-presenting cells, mainly DCs, present salivary immunogens to lymphocytes, which trigger cell-mediated and antibody responses (22). Sets of immunoglobulins and long-lived memory T and B cells are induced in the host (Figure 3). In subsequent contacts with the same tick, acquired immunity is quickly activated to yield a more specific protective response. It has been reported that tick saliva suppresses adaptive immunity in various ways (33). Some tick salivary compounds interfere with DCs and alter their capacity to present antigens. Other compounds prevent lymphocyte proliferation or T cell cytokine production.

#### Antigen Processing and Presentation

Several tick salivary molecules target DCs, thereby preventing the initiation of adaptive immunity. PGE_2_ has been described as the major DC inhibitor (Figure 3) in both *I. scapularis* (130) and *Amblyomma sculptum* saliva (131). RHS2 from *Rhipicephalus haemaphysaloides* can inhibit the differentiation of BMDCs into DCs *in vitro* and promote their differentiation into macrophages (132). RHS2 also inhibits the expression of CD80, CD86, and the major histocompatibility complex class II (MHCII), thereby preventing DC maturation. DC differentiation from monocytes is blocked by Japanin (Figure 3), a salivary gland lipocalin from *R. appendiculatus* (133). Moreover, Japanin was found to reprogram DC responses to a broad variety of stimuli *in vitro*, radically altering the expression of co-stimulatory and co-inhibitory molecules and modifying the production of diverse cytokines (134).

#### Humoral Immunity

Tick salivary components also disarm host humoral immunity by inhibiting B cells responses, including their production of specific anti-tick antibodies (134). BIP (B cell inhibitory protein) (Figure 3) derived from *I. ricinus* inhibited B lymphocyte proliferation induced by *B. burgdorferi* lipoproteins (135). Similarly, BIF (B cell inhibitory factor) from *H. asiaticum asiaticum* salivary glands (Figure 3) was found to suppress B cell responses (136).

#### Cell-Mediated Immunity

Numerous inhibitors of T cell functions have been identified in tick saliva and salivary glands. Three immunosuppressant salivary proteins, p36 from *Dermacentor andersoni* (137, 138), HL-p36 from *H. longicornis* (139), and RH36 from *R. haemaphysaloides* (140) suppressed T lymphocyte proliferation *in vitro*. Recombinant HL-p36 and RH36 also directly inhibited the proliferation of several mitogen-stimulated cells *in vivo* and the expression of several cytokines such as IL-2, IL-12, and TNF-α (141, 142). In addition to its effect on DCs, RHS2 can prevent the activation of CD4^+^ and CD8^+^ T cells, leading to inhibition of the Th1 immune response (132). Two salivary cystatins from *I. scapularis*, Sialostatin L and Sialostatin L2, have shown promising anti-inflammatory and immunosuppressive activity *in vitro* and *in vivo* (141, 142). Sialostatin L was immunosuppressive in mammalian models of immune-related diseases, suppressing the proliferation of both CD4 and CD8 T cells, neutrophil migration in severe inflammation, and the secretion of cytokines by MCs, DCs, and lymphocytes (130, 141). Sialostatin L reduced the release of IL-9 by Th9 cells, which might explain its suppression of airway hyperresponsiveness and eosinophilia in an experimental asthma model (143). In the experimental autoimmune encephalomyelitis (EAE) mouse model of multiple sclerosis, *in vivo* administration of Sialostatin L significantly prevented disease symptoms (144). Furthermore, Sialostatin L effectively altered lysosomal cysteine cathepsins L, C, V, S, X and papain activity, critical elements in antigen presentation (141). Sialostatin L2 suppressed IFN-β-mediated immune reactions in murine DCs (145), and was also found to inhibit cathepsins C, L, S, and V and decrease IL-1β and IL-18 secretion by macrophages (146). Similar to sialostatins, Iristatin, a novel type 2 cystatin from *I. ricinus*, inhibited the proteolytic activity of cathepsins L and C (147) and diminished IL-2, IL-4, IL-9, and IFN-γ production by different T cell populations, IL-6 and IL-9 production by MCs, and nitric oxide production by macrophages. Iristatin inhibited OVA antigen-induced CD4^+^ T cell proliferation and leukocyte recruitment *in vivo* and *in vitro* (Figure 3). With such a wide spectrum of immunosuppressive activities, Iristatin may be exploitable as an immunotherapeutic.

T cell inhibitory salivary molecules also include Iris and IrSPI from *I. ricinus* (148), Ipis-1 from *I. persulcatus* (149), RmS-3 and RmS-17 from *R. microplus* (61), and Salp15 from *I. scapularis* (47). Iris was found to suppress T cell proliferation, promote a Th2-type response, and bind to monocytes/macrophages, inhibiting their ability to secrete pro-inflammatory cytokines IL-6 and TNF-α (150). Similarly, IrSPI suppressed proliferation of CD4^+^ T lymphocytes and pro-inflammatory cytokine secretion (Figure 3) from splenocytes and macrophages (151). The Iris homolog Ipis-1 inhibited cellular proliferation and the production of IFN-γ in bovine peripheral blood mononuclear cells, suggesting that Ipis could directly interact with T cells and inhibit their functions (149). RmS-3 and RmS-17 inhibited the metabolic activity of lymphocytes, decreasing lymphocyte proliferation (61). One of the most extensively studied components of tick saliva is Salp15, an immunosuppressive cysteine-rich glycoprotein (47). Salp15 binds specifically to CD4 co-receptors on the surface of T lymphocytes, interfering with TCR-mediated signaling transduction and impeding IL-2 secretion in a dose-dependent manner (47, 148, 149). A subsequent study reported that Salp15 interacts with DC-SIGN on DCs, triggering a novel Raf-1/MEK-dependent signaling pathway, thereby impairing cytokine production and T cell proliferation (152). Apart from its effect on adaptive immunity, Salp15 inhibited TLR2-dependent keratinocyte inflammation *in vitro* (153). Together, these findings make this salivary protein a potential candidate for the treatment of T cell-mediated autoimmune diseases. Indeed, the immunomodulatory effect of Salp15 has been investigated in various models *in vivo*. Paveglio and colleagues (154) were the first to show that Salp15 has a therapeutic effect in a mouse model of allergic asthma. The specific binding of Salp15 to CD4 inhibited the proliferation and differentiation of CD4^+^ T cells toward Th2 cells and suppressed the production of inflammatory cytokines, significantly reducing symptoms of allergic asthma in treated mice. Moreover, Salp15 prevented the association of HIV-1 gp120 and CD4 in an experimental HIV infection (155). gp120 is an HIV-1 envelope glycoprotein which, by binding to CD4, promotes penetration of HIV-1 into the host cell (156). The authors suggested that Salp15 competes with gp120 for an association with CD4 due to its ability to interact with CD4 on T cells. Another investigation showed the opposite effect in murine EAE/multiple sclerosis (157). The occurrence and progression of EAE is associated with myelin-specific CD4^+^ T cell activation and secretion of IL-17 and INF-γ by Th17 and Th1 cells, respectively (158). Unexpectedly, Salp15 worsened the pathology in mice upon induction of EAE. A possible reason for this might be that Salp15 promoted Th17 activation, increasing IL-17 levels *in vivo*, and it also enhanced Th17 differentiation in the presence of IL-6 and absence of TGF-β *in vitro* (157). More recently, it was shown that Salp15 binding to CD4 is persistent and induces a long-lasting immunomodulatory effect in a murine model of hematopoietic transplantation (159). Based on these results, Salp15 could provide important opportunities for the development of novel and sophisticated therapies for human disease mainly those mediated by multiple processes.

## Tick Non-Coding RNAs: New Alternatives in Targeted Immunomodulatory Therapy?

**FIGURE 4:**
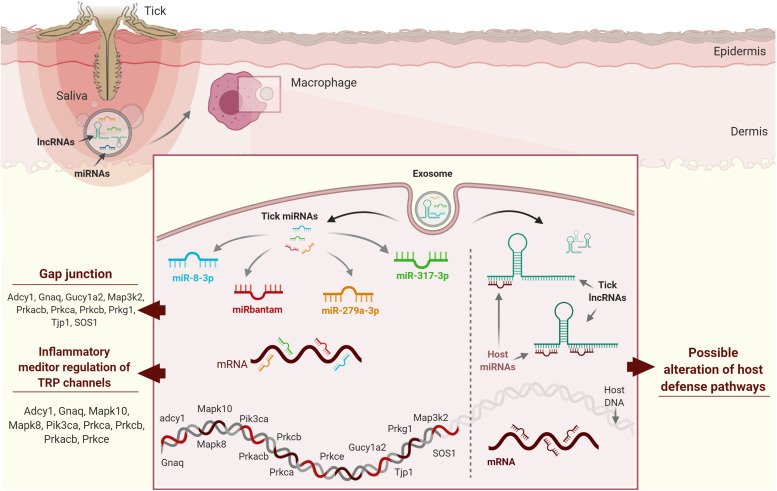
Possible strategies used by tick ncRNAs in host cells. (A) miR-8-3p, bantam-3p, mir-317-3p, and miR-279a-3p from *Ixodes ricinus* have been predicted to target gap junctions and inflammatory mediator regulation of TRP channels, which play a role in host homeostatic responses. (B) Host miRNAs regulate protein coding genes through mRNA cleavage and/or direct translational repression and/or mRNA destabilization. Ticks secrete exosomes in their saliva containing lncRNAs, which may compete with host mRNAs for host miRNA binding by acting as “sponge” molecules that inhibit host miRNA interactions with host mRNAs, thus affecting host homeostatic responses to tick feeding.

Tick salivary molecules are pluripotent (i.e., one molecule may affect more than one host cell population) and show considerable functional redundancy (i.e., the same host cell population may be targeted by more than one tick salivary molecule) (160). Tick saliva not only contains proteins and metabolites but also non-proteinaceous species including nucleic acids (22, 161) and RNA silencing signals, which are now recognized as crucial for cross-species communication across diverse biological niches (162, 163). With respect to the tripartite tick-pathogen-host interaction, recent transcriptomic and proteomic projects, facilitated by rapid developments in high-throughput sequencing, have revealed several tick genes/transcripts of unknown function or transcripts without an open reading frame (ORF) (160). These projects have also revealed a significant number of ncRNAs in tick saliva that are predicted to be functionally involved in the vector-host interaction (164). ncRNAs are important in vector-host interactions at the tick-host interface: ticks use ncRNAs, broadly divided by length into long non-coding RNAs (lncRNAs) (>200 nucleotides (nt) long) and small non-coding RNAs (sncRNAs) (<200 nt long), to hijack host defense mechanisms (165). They are suggested to be secreted via exosomes (166, 167) and released in the host cells, to manipulate gene expression and deregulate host defense pathways (165). LncRNAs are not translated into proteins and include mRNA-like intergenic transcripts (lincRNAs), antisense transcripts of protein-coding genes, and primary RNA polymerase II (Pol II) transcript-derived unconventional lncRNAs (168). LncRNAs are involved in numerous important biological phenomena such as imprinting genomic loci, shaping chromosome conformation and allosterically regulating enzymatic activity (169). On the other hand, sncRNAs represent a diverse set of molecules that control the expression of most vertebrate genes and include small-interfering RNAs (siRNAs), microRNAs (miRNAs), and piwi-interacting RNAs (piRNAs) (170). miRNAs have been extensively studied in several organisms and their function in gene regulation is well understood. The first attempt to identify host targets of tick ncRNAs was performed by Hackenberg and colleagues (171). Their *in silico* study showed that saliva-specific miRNAs from *I. ricinus* might have combinatorial effects on the host targetome: different tick miRNAs may target vertebrate host genes in the same host homeostatic pathway, and the expression of a given host gene may also be regulated by more than one saliva-specific tick miRNA (171). The authors suggested combinatorial effects of vector miRNAs on host target genes, which may be of importance in evolutionary terms to maintain robust regulation of host genes and pathways important in the tick-host interaction. miR-8-3p, bantam-3p, mir-317-3p, and miR-279a-3p from *I. ricinus* were predicted to target host KEGG pathways such as “gap junction” and “inflammatory mediator regulation of TRP channels,” which play a role in the host homeostatic response (Figure 4A).

The huge number of ncRNA sequences revealed by high-throughput projects suggests that tick ncRNAs might also modulate host gene expression by binding to regulatory host miRNAs involved in host immune cell responses. Tick ncRNAs could regulate protein-coding genes through mRNA cleavage and/or direct translational repression and/or mRNA destabilization. We hypothesize that lncRNAs compete with host mRNAs for host miRNA binding by acting as “sponge” molecules that inhibit host miRNA interactions with host mRNAs, thus affecting host homeostatic responses to tick feeding (Figure 4B). *In silico* predictions now need to be validated using systems-based approaches in order to characterize tick ncRNAs and to understand their involvement in tick-host interactions. Various academic and commercial research groups are now exploring ncRNA-based therapies and exploring the potential of miRNA therapeutics, as miRNAs are the most studied ncRNAs to date (172). A few miRNAs have entered preclinical and clinical testing, so might soon be available on the market for use in humans. As tick ncRNAs are likely to be less immunogenic by their intrinsic ability to hijack and bypass host immunity, we strongly believe that this flourishing field might lead to ncRNA therapeutics useful for the treatment of various diseases. Emerging data on the possible regulatory function of ncRNAs, the vector, the pathogen, and the host, the role of ncRNA in the vector or host-pathogen remain quite challenging, due to several biological and technical aspects. For example, the used techniques for ncRNAs study can generate several biases in the results. New scRNA-seq technology that is showing its efficiency to counter this issue, but sufficient read depths of rarely expressed RNAs can also be misleading, and vector, host or pathogen-derived RNAs information could be easily lost. LncRNAs data analyses also remain controversial due to the lack of accurate transcript models in current annotations. LncRNAs could also be pluripotent, and just as the salivary proteins, this would also be a serious limitation in any potential therapeutic applications. Overall, given the arising data on the ability of ncRNAs produced in tick salivary glands and also the possible regulatory function of cellular RNAs on the vector, pathogen or host, the role of ncRNA in the vector or host-pathogen crosstalk might be even more sophisticated than what is expected. Development of research projects that employ single-cell/single molecule sequencing methodologies and do not require nucleic acid amplification will definitely enable less biased and reliable data output.

## Concluding Remarks

Inflammatory, autoimmune, metabolic, and neurodegenerative diseases remain critical health problems worldwide, creating an enormous need for new, effective, and specific medicines. Over the last few decades, ticks have shown their “good side” as promising sources of new drugs targeting distinct pathophysiological pathways in mammals. In this review, we summarized potential drug candidates from tick salivary glands that might have therapeutic use in immune disorders and other related diseases. We have also highlighted that tick ncRNAs are possible new alternatives for targeted immunomodulatory therapy. The molecules described in this review have been tested *in vitro* and/or in various animal models of human disease and have shown encouraging results. However, only a few of them have advanced to (pre-)clinical investigations, and further in-depth molecular and cellular studies are required to further develop candidates for clinical trials. The major challenges to drug development are (i) specificity and efficacy, since broad-spectrum targets can generate unwanted side-effects; and (ii) safety, with drug-induced toxicity and immunogenicity critical limitations of many drug candidates. Addressing these challenges, tick salivary molecules, at least in theory, might have a somewhat higher clinical success rate than molecules from other origins, since many tick-derived products have an intrinsically low risk of toxicity and immunogenicity and high target specificity to execute their intended functions. From the pharmaceutical perspective, some molecules might be unstable or have a short half-lives, so pharmacokinetic, dose optimization, and modification studies are critical research priorities. While experimental validation and ultimately clinical trialing remain the cornerstone of successful therapeutic development, computational methods might play a role as time-saving, cost-effective, and productive approaches to design and discover novel lead compounds for associated disease targets. Modeling and simulation of preclinical and clinical trials could also be used to bridge the gap between the early stages of drug development and their potential effects on humans.

## Author Contributions

All authors searched and read the literature and edited the manuscript. HA, CB, and MK wrote the manuscript.

## Conflict of Interest

The authors declare that the research was conducted in the absence of any commercial or financial relationships that could be construed as a potential conflict of interest.

## Abbreviations

AAS: *Amblyomma americanum* serpin
BIF: B cell inhibitory factor
BIP: B cell inhibitory protein
BMDCs: mouse bone marrow-derived dendritic cells
BMDMs: mouse bone marrow-derived macrophages
CD: cluster of differentiation
CFA: complete freund’s adjuvant
DC: dendritic cells
EAE: experimental autoimmune encephalomyelitis
HIV: human immunodeficiency viruses
HL-p36: *Haemaphysalis longicornis* p-36
IFN-γ: interferon gamma
IL: interleukin
Irac: *Ixodes ricinus* anticomplement
IRS-2: *Ixodes ricinus* Serpin- 2
Isac: *Ixodes scapularis* salivary anticomplement
LPS: lipopolysaccharide
MBL: mannose-binding lectin
MC: Mast cells
MCP-1: monocyte chemoattractant protein 1
MHC-I, -II: major histocompatibility complex-I, -II
MIF: macrophage migration inhibitory factor
MS: mass spectrometry
NGS: next-generation sequencing
OmCI: *Ornithodoros moubata* complement inhibitor
PDAC: pancreatic ductal adenocarcinoma
PGE_2_: prostaglandin E2
Ra-HBPs: *Rhipicephalus appendiculatus* histamine-binding proteins
RmS-3: *Rhipicephalus microplus* serpin 3
Salp: salivary protein
SHBP: serotonin and histamine-binding protein
TCR: T cell receptor
TdPI: Tick-derived protease inhibitor
TGF-β: transforming growth factor-beta
Th: T helper
TLR: toll-like receptors
TNF: tumor necrosis factor
TSLPI: tick salivary lectin pathway inhibitor.
